# The XRE family protein DbuR is a transcriptional repressor of the *dbu* operon in *Pseudomonas putida*

**DOI:** 10.1128/aem.01715-25

**Published:** 2025-11-25

**Authors:** Ronnie L. Fulton, Diana M. Downs

**Affiliations:** 1 Department of Microbiology, University of Georgia 1355, Athens, Georgia, USA; Washington University in St. Louis, St. Louis, Missouri, USA

**Keywords:** *Pseudomonas putida*, DbuR, D-branched chain amino acids, XRE regulators

## Abstract

*Pseudomonas putida* is a ubiquitous, metabolically versatile microbe that has gained visibility as a chassis for bioengineering. The *dbu* operon in *P. putida*, comprised of a D-branched-chain amino acid (D-BCAA) oxidase (DbuA), a Rid2 protein (DbuB), and a transporter (DbuC), was previously characterized for its role in the catabolism of D-branched-chain amino acids. In the present study, we show that the addition of any of three D-BCAAs catabolized by these gene products increases expression of the *dbu* genes. The presence of D-tyrosine does not increase transcription of the *dbu* genes, nor is it used as a sole nitrogen source by *P. putida*. Derepression of the *dbu* operon allows catabolism of D-tyrosine, thus uncovering an additional capacity of the *dbu* gene products. Results herein show that the *dbuR* gene, which is divergently transcribed from the operon, encodes a xenobiotic response element (XRE) family transcription factor that is a transcriptional repressor of the *dbu* genes. The effect of D-BCAA on *dbu* expression appears to be at least partially independent of DbuR, suggesting transcriptional regulation of this operon involves multiple components. In total, this work contributes to understanding the complex regulatory and metabolic networks of the environmentally and economically important microbe *P. putida*.

**IMPORTANCE:**

*Pseudomonas putida* is a broadly utilized bioengineering chassis, primarily due to the versatile and robust metabolic network of the organism. *P. putida*, like many microorganisms, employs a complex regulatory network to coordinate its metabolism, allowing it to adapt to changing environments and dynamic nutrient availability. The physiological role of the D-branched-chain amino acid (D-BCAA) utilization pathway (*dbu*) operon in D-BCAA metabolism by *P. putida* has been defined, and herein we provide insights into the regulation of the genes in this operon. DbuR is encoded by a gene near the *dbu* operon, and this protein represses the expression of the *dbu* genes. A new metabolic capability (catabolism of D-tyrosine) of *P. putida* was revealed, a capability that increases the potential of this organism as a chassis for bioengineering. This work expands current knowledge of *P. putida* and contributes insights into the metabolic and regulatory capabilities of this environmentally, industrially, and economically relevant microbe.

## INTRODUCTION

Microbes occupy diverse ecological niches where they encounter a variety of changing nutritional and environmental conditions (1–3). To thrive under dynamic nutritional conditions, many microbes contain genes encoding situationally relevant metabolic pathways. Expression of these genes and activity of the gene products can be appropriately regulated to create a metabolic state that is efficient in the relevant environmental niche. The pattern of transcriptional regulation of genes can provide insight into their function and physiological role, often suggesting an environmental niche where the genes contribute to fitness. Thus, deciphering the regulatory mechanisms of metabolic pathways can inform efforts to model the metabolic capabilities of clinically, environmentally, and industrially relevant organisms.

*Pseudomonas putida* is an industrially relevant microbe that has been recognized as a chassis for diverse bioengineering efforts due to its versatile metabolism and facile genetic tractability (4–7). The importance of *P. putida* as a foundation for metabolic engineering, along with its ubiquity in the environment, underscores the need to better define the metabolic pathways used by this organism, and the regulatory mechanisms applied to coordinate these pathways. The genome of *P. putida* encodes multiple pathways that catabolize diverse, often non-canonical, carbon and nitrogen sources. Among these is the *dbu* operon.

A physiological role for the *dbu* (D-branched-chain amino acid utilization) operon in *P. putida* has been described (8). The gene products encoded in this operon constitute a pathway that allows *P. putida* to utilize certain D-branched-chain amino acids (D-BCAAs) as a source of carbon and/or nitrogen. The gene products include a D-amino acid oxidase (DbuA), a Rid2 family protein (DbuB), and a transporter (DbuC) (8). D-BCAAs are presumed to be transported by DbuC, prior to being oxidized about the alpha carbon by DbuA to generate an imine intermediate. The resulting imines are deaminated to the corresponding branched-chain ketoacid (BCKA) and ammonia; either spontaneously by water or more rapidly, by the Rid protein DbuB. The resulting ammonia serves as a source of nitrogen, while the BCKAs can be converted to short-chain acyl-CoAs and enter central carbon metabolism to serve as a carbon source. Alternatively, the keto acids can be acted on by a transaminase to generate L-BCAA building blocks.

Upstream, and divergently transcribed from, the *dbu* operon, is a gene encoding an annotated xenobiotic response element (XRE)-like/Cro/CI family transcriptional regulator (DbuR). Precedent and genomic position suggested that DbuR could regulate expression of the *dbu* operon. This study was initiated to test this possibility. Results herein confirm that DbuR is a transcriptional repressor of the *dbu* operon in *P. putida*. Data further show that the presence of D-BCAAs in the medium increased transcription of the *dbu* genes, seemingly by a DbuR-independent mechanism. Finally, this work identified a new role for the *dbu* pathway with the demonstration that D-tyrosine can be used as the sole nitrogen source when the *dbu* genes are overexpressed.

## RESULTS AND DISCUSSION

### DbuR is a transcriptional repressor of the *dbu* locus in *P. putida*

**Fig 1:**
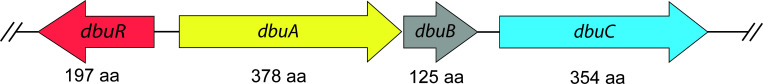
Schematic of the *dbu* locus. Shown are the genes of the *dbu* operon that encode—a D-amino acid oxidase (*dbuA*, yellow), a Rid2 protein (*dbuB*, gray), a transporter (*dbuC*, blue), and the XRE/Cro/CI family transcriptional regulator (*dbuR*, red). The promoter element for *dbuABC* was predicted to lie in the 114 bp between *dbuR* and *dbuA* using the BPROM online tool (13). The number of amino acids in the encoded proteins is indicated below the genes.

The *dbu* operon in *P. putida* contains genes encoding a D-BCAA oxidase (DbuA), a Rid2 subfamily protein (DbuB), and an annotated ABC transporter (DbuC) (Fig. 1) (8). Adjacent to the *dbu* operon and divergently transcribed is *dbuR,* encoding a protein annotated as an XRE-like/Cro/CI family transcriptional regulator. DbuR is predicted to contain both helix-turn-helix and cupin signal-sensing domains (Fig. S1) (9–11). Proteins of the (XRE)-like/Cro/CI family commonly repress transcription of genes adjacent to the gene encoding them. Often, the genes adjacent to the regulator encode metabolic pathways that are relevant in a specific niche or nutritional condition (12). A *lacZ* transcriptional fusion was constructed in the chromosome to determine the effect of DbuR on the transcription of the *dbu* operon. The *dbuA* gene was replaced with *lacZ* (*dbuA::lacZ*), making β-galactosidase activity a proxy for expression of the *dbu* operon. β-galactosidase activity was assayed in strains containing a *dbuA::lacZ* with or without a functional *dbuR* gene. The two relevant strains were grown in minimal glucose medium, and β-galactosidase activity was quantified when the cultures reached the early exponential phase (OD_600_ = 0.3–0.5). The strain lacking *dbuR* had more than 15-fold higher β-galactosidase activity than the strain carrying the wild-type gene (1,154 ± 44 and 57 ± 9, respectively). The simple interpretation of these data is that DbuR is a repressor of the *dbu* operon, and transcription is induced in its absence.

### DbuR binds and protects sequences between *dbuR* and the *dbuA*

**Fig 2:**
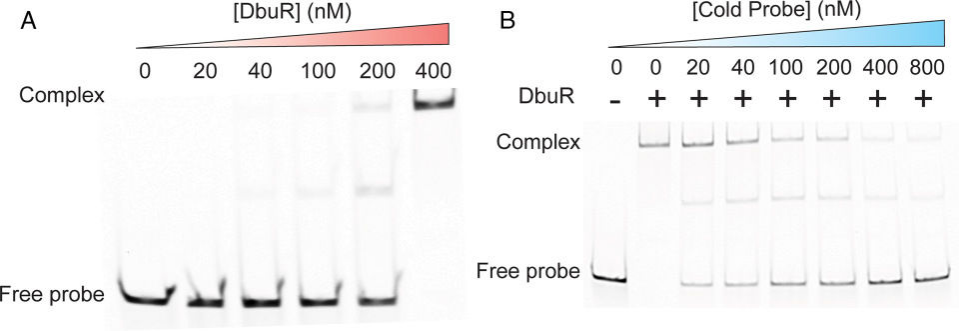
DbuR binds the region between *dbuR* and *dbuA*. (**A**) The 133 bp fragment (20 nM) described in the text was incubated with the indicated concentration of purified DbuR prior to separation by polyacrylamide electrophoresis. The 5′ 6-carboxyfluorescein (5′ 6-FAM) labeled probe was generated by PCR amplification of the intergenic region between *dbuR* and *dbuA*, with a 5′ 6-FAM labeled forward primer and an unlabeled reverse primer. (**B**) Electromobility shift experiments were repeated with reactions that contained labeled probe (20 nM) and DbuR (400 nM). An unlabeled probe was added at the indicated concentration prior to electrophoresis. All experiments were repeated at least three times with comparable results.

DbuR protein was purified to >95% homogeneity (Fig. S2) and tested for direct binding to sequences upstream of the *dbu* operon. A DNA fragment that included nucleotides of the intergenic region (114 bp) and 19 bp into the *dbuA* coding sequence was generated. The resulting 133 bp fragment was presumed to contain the *dbu* promoter and was used as a probe in molecular experiments. Initially, the DNA probe (20 nM) was incubated with increasing concentrations of DbuR prior to being subjected to polyacrylamide electrophoresis (Fig. 2). The mobility of the DNA probe decreased as a function of increasing DbuR concentration. When DbuR was present in a 20-fold molar excess (20 nM probe, 400 nM DbuR), the movement of the probe was greatly decreased, with a single low mobility band detected (Fig. 2A). At lower molar ratios of DbuR to probe (i.e., ~5:1), a second band of intermediate mobility became visible. The presence of a species of intermediate mobility has been seen in other systems and suggests that DbuR binds the probe with different stoichiometry at lower molar ratios (14). Unlabeled probe competed with the fluorescently labeled probe, restoring the free labeled probe as indicated by the high mobility band (Fig. 2B). In total, these data supported the hypothesis that DbuR binds the intergenic region between the *dbuR* gene and the *dbuA* gene.

**Fig 3:**
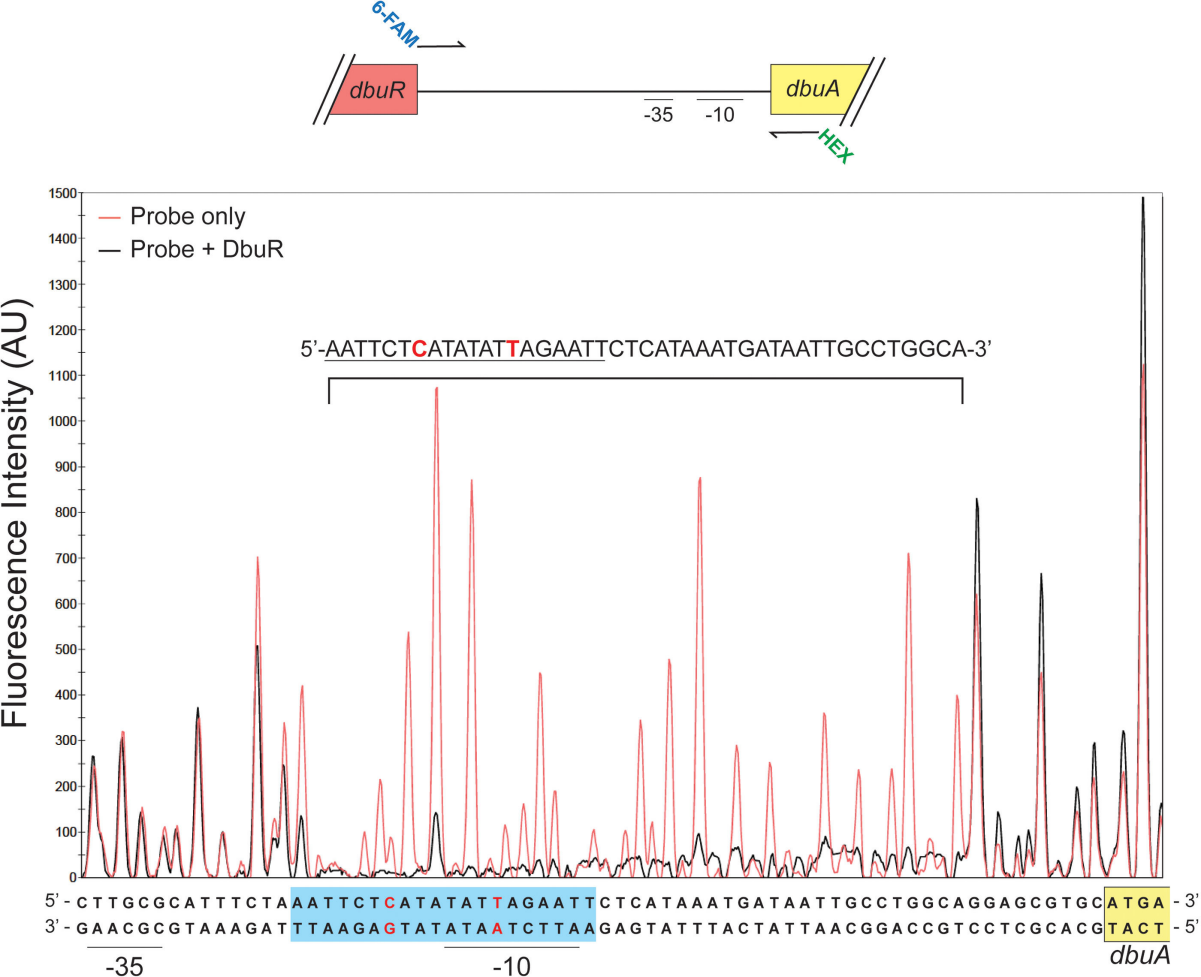
DbuR binds a 44 nt sequence upstream of *dbuA*. The 5′ 6-FAM (green) and 5′-hexachlorofluorescein (HEX, blue) labeled fragment used in DNase I footprinting experiments is shown schematically above the electropherogram. The labeled DNA probe (20 nM) was digested by DNase I after incubation with (200 nM, black) or without DbuR (red). Peaks were compared to Sanger sequencing of the *dbu* promoter region. Protected nucleotides are shown above the relevant peaks on the electropherogram. A 20 bp palindromic sequence was noted and is underlined. Also shown is a schematic depicting the positions of the palindromic sequence (blue boxes) relative to the promoter elements (−10 element and −35 element) and the start of the coding sequence of *dbuA* (yellow). Red-colored bases show the imperfect base pairs of the palindromes. The electropherogram depicts fragments detected using the 6-FAM label (i.e., top strand). The electropherogram depicting HEX-labeled (i.e., bottom strand) DNA is shown in Fig. S3. There was no protected region upstream of what is shown above. Experiments were performed in technical duplicate with comparable results.

The sequence bound by DbuR was determined by DNase I footprinting analyses with the 133 bp DNA probe used in the electromobility shift assays (EMSAs) above. Figure 3 shows the results for fragment analysis of DNase I digestions of the coding strand of the *dbu* operon after the probe was incubated with (black) or without DbuR (red). The sequence of the protected region was determined by aligning electropherograms of DNase I digestion reactions to those obtained from Sanger sequencing (Fig. S3A). The data showed that when DbuR was present, a 44 bp region was protected from DNase I digestion. The sequence that was protected included a 20 bp palindrome (5′-AATTCTCATATATTAGAATT-3′) with one imperfect base pair. The sequence protected by DbuR included the sequence predicted by the BPROM online tool to be the −10 element of the *dbu* promoter (13). The position of the protected sequence in the context of *dbuA* and *dbuR* is shown in Fig. S4. This palindrome is similar in size (i.e., 15–25 bp), sequence, and promoter proximal location (i.e., −20 and +10 bp of the promoter) to binding sites reported for other XRE-cupin regulators, including NceR of *Neisseria gonorrhoeae*, and several regulators in *P. aeruginosa*—i.e., PsdR, ErfA, and PauR (12, 15, 16). Like those above, these data are consistent with DbuR being a direct repressor of the *dbu* genes.

### DbuR is not the sole regulator of the *dbu* operon

The *dbu* operon is required for *P. putida* to utilize some D-branched-chain amino acids (D-BCAAs) as the sole nitrogen source (i.e., D-leucine, D-isoleucine, D-valine) (8). In a simple scenario, one or more of these D-BCAAs would serve as an effector of DbuR and relieve DNA binding to allow transcription of the *dbu* operon when catabolism of the amino acid is beneficial. Strains carrying a *dbuA::lacZ* fusion with or without a functional DbuR (DMPA223 or DMPA224, respectively) were grown in minimal glucose medium. Ammonium chloride was in the medium as a nitrogen source, and D-leucine, D-isoleucine, or D-valine (10 mM) was added. β-galactosidase activity was quantified in the two strains for each condition (Table 1). Two points were noted from the data. First, β-galactosidase activity was increased more than 10-fold by each of the D-BCAAs in strain DMPA223 (*dbuR*+). D-isoleucine resulted in the biggest increase of ~40-fold. Secondly, contrary to the expectation of a simple model, the D-BCAAs increased β-galactosidase activity with or without a functional DbuR, to a level that was specific to the respective D-BCAA (Table 1). This result showed that the effect of the D-BCAAs could not be simply explained by eliminating the binding of DbuR and suggested that this protein was not solely responsible for the regulation of *dbu* transcription. The above data were consistent with a model in which there is an activator(s) of *dbu* that requires a D-BCAA(s) as a co-effector. In such a model, the expression of *dbu* would be repressed by DbuR in the absence of D-BCAAs, while in the presence of D-BCAAs, the activator complex would compete with DbuR for binding. A caveat with these results is that the catabolism of the provided D-BCAAs was blocked by the disruption of *dbuA*.

**TABLE 1:** D-branched-chain amino acids increase transcription of the *dbu* locus^*a*^

Addition	β-galactosidase activity (Miller units) for strain:	
	DMPA223 (*dbuR*^+^)	DMPA224 (*dbuR*^−^)
None	57 ± 9	1,154 ± 44*
D-leucine	658 ± 42	793 ± 72
D-isoleucine	2,684 ± 40	2,812 ± 101
D-valine	753 ± 71	838 ± 67

^*a*^ Strains DMPA 223 (*dbuA*::*lacZ*) and DMPA 224 (Δ*dbuR dbuA*::*lacZ*) were assayed for β-galactosidase activity as described in Materials and Methods. Where indicated, D-BCAAs (10 mM) were in minimal glucose (11 mM) medium with ammonium chloride (10 mM) as a nitrogen source. Results are the mean and standard deviation between three independent biological replicates. Statistical significance was determined by Sidak’s multiple comparisons test. A significant difference (*P* ≤ 0.05) in β-galactosidase activity between the two strains was only in the medium with no additions, as denoted by an asterisk.

### Derepression of the *dbu* operon allows D-tyrosine to be used as the sole nitrogen source

**Fig 4:**
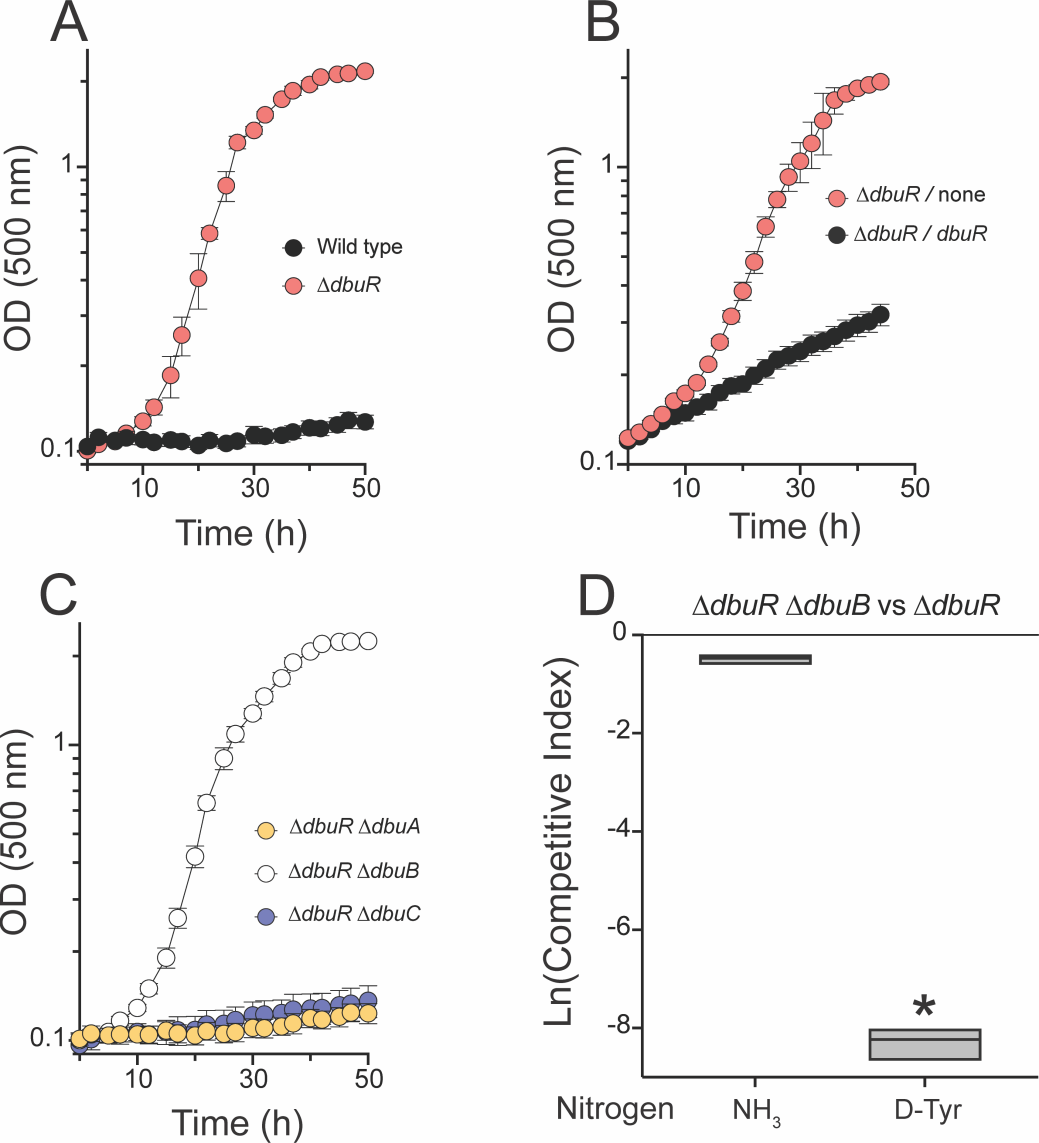
The *dbu* locus is implicated in D-tyrosine catabolism. (**A**) Growth profiles of indicated strains are shown in minimal glucose (11 mM) medium with D-tyrosine (5 mM) as the sole nitrogen source—wild type (black) and ∆*dbuR* (red). (**B**) Growth of ∆*dbuR* strains with the *rhaBAD* promoter with no gene downstream (red) or expressing *dbuR* (black). Strains were grown in minimal glucose medium with D-tyrosine as the sole nitrogen source supplemented with 5 mM rhamnose. (**C**) Growth of *P. putida* strains in minimal glucose medium with D-tyrosine as the sole nitrogen source*—∆dbuR ∆dbuA* (orange), *∆dbuR ∆dbuB* (white), and *∆dbuR ∆dbuC* (purple). (**D**) Competitive indices of a *∆dbuR ∆dbuB* double mutant (DMPA231) grown in competition with the ∆*dbuR* parent strain (DMPA199) in minimal glucose medium with ammonia (10 mM) or D-tyrosine (5 mM) as the sole source of nitrogen. An asterisk denotes a significant difference in CI values, as determined by Student’s *t*-test, compared to control experiments in which two *∆dbuR ∆dbuB* strains (DMPA231 and DMPA232) were competed under the same conditions. Error bars represent the standard deviation between three independent biological replicates.

Wild-type *P. putida* cannot utilize D-tyrosine as the sole nitrogen source for growth. Unexpectedly, strains lacking DbuR gained the ability to catabolize D-tyrosine (Fig. 4A). When *dbuR* was expressed ectopically in the *att*Tn7 site under control of a rhamnose-inducible promoter, growth of the Δ*dbuR* strain on D-tyrosine was decreased to levels near those of wild type (Fig. 4B). This result supported the causality of the Δ*dbuR* mutation, while the weak growth of the merodiploid strain suggested that the expression of *dbuR* from the rhamnose promoter was lower than at its native site.

The data in Fig. 4 suggested that catabolism of D-tyrosine for growth required increased expression of one or more of the *dbu* genes. Deletion mutations in *dbuA*, *dbuB,* or *dbuC* were introduced in a Δ*dbuR* genetic background, and the resulting three strains were assessed for growth with D-tyrosine as the sole nitrogen source. The Δ*dbuR* strains lacking DbuA or DbuC did not grow with D-tyrosine as a nitrogen source. In contrast, the strain lacking DbuB grew, as well as the parental Δ*dbuR* mutant (Fig. 4C). Despite having no fitness defect in monoculture, the double mutant (Δ*dbuR* Δ*dbuB*) had a significant competitive disadvantage (Ln(CI) of −8 corresponding to a ~2,900-fold decrease in competitive fitness) in co-culture with the parental Δ*dbuR* mutant when D-tyrosine was the sole nitrogen source (Fig. 4D). These results paralleled those reported for the role of the *dbu* gene products in the catabolism of other substrates of the pathway encoded by the *dbu* operon (8).

**Fig 5:**
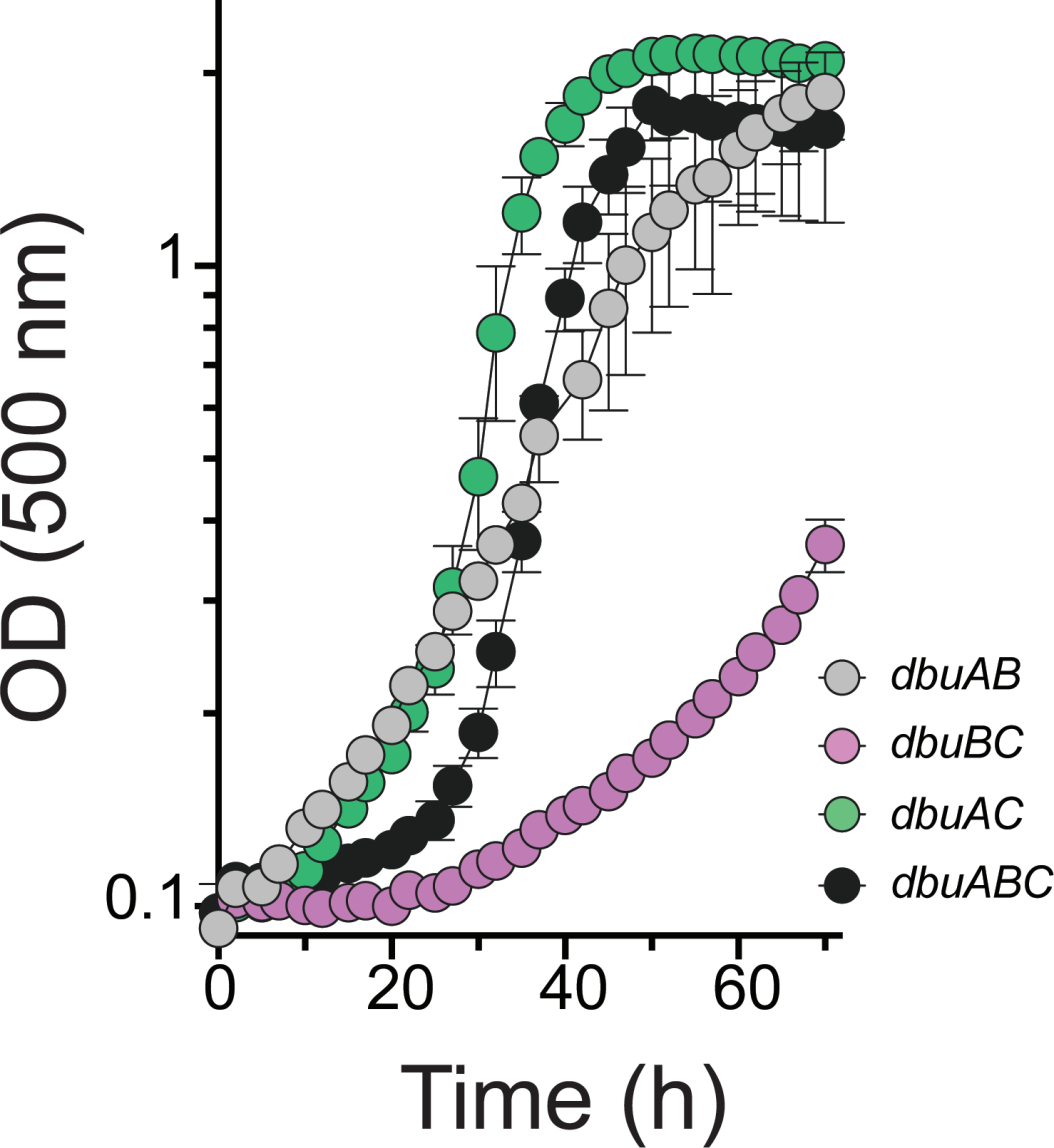
Overexpression of *dbu* genes allows D-tyrosine utilization by *P. putida*. Shown are growth profiles of *P. putida* wild-type strains with the *dbu* genes indicated in the legend, inserted into the attTn7 site under the control of the *PrhaBAD* promoter. Strains shown are DMPA219-222. Strains were grown in minimal glucose (11 mM) medium with D-tyrosine (5 mM) as the sole nitrogen source and supplemented with 5 mM rhamnose. Data and error bars represent the mean and standard deviation between three independent biological replicates.

The role of the *dbu* locus in D-tyrosine utilization was confirmed with analysis of strains expressing various combinations of the *dbu* genes under control of a rhamnose-inducible promoter, in addition to carrying the operon in its native location. Strains were grown in minimal medium with D-tyrosine provided as the sole nitrogen source and L-rhamnose (5 mM) added to induce expression of the respective genes (Fig. 5). Of the single gene merodiploids, only the one expressing *dbuA* grew with D-tyrosine as the sole nitrogen source (data not shown). Consistently, strains expressing *dbuA* in addition to *dbuC*, *dbuB,* or all of the *dbu* genes also exhibit growth profiles similar to that of a Δ*dbuR* mutant. Unexpectedly, the strain expressing *dbuB* and *dbuC* had significant growth with D-tyrosine. This result suggests that increased levels of DbuB and DbuC facilitate the ability of chromosomally expressed levels of *dbuA* to mediate growth with D-tyrosine.

### D-BCAAs stimulate the catabolism of D-tyrosine

The catalysis of D-tyrosine was shown in two non-physiological situations: (i) when strains lack *dbuR* or (ii) one or more *dbu* genes were ectopically expressed. Since D-tyrosine did not induce expression of the *dbu* genes (data not shown), the physiological relevance of the ability to catabolize this amino acid was unclear. Additional experiments found a situation in which D-tyrosine catabolism could contribute to growth. In the presence of low concentrations of D-leucine, D-valine, or D-isoleucine, wild-type *P. putida* used D-tyrosine as a nitrogen source. When provided at 125 µM as the sole nitrogen source, the individual D-BCAAs did not support growth (Table 2). However, the addition of D-tyrosine (5 mM) in this medium resulted in growth equivalent to that allowed with ammonium as the sole nitrogen source. These data are consistent with a scenario in which the D-BCAAs induce expression of the *dbu* operon and allow the catabolism of D-tyrosine to be the primary nitrogen source. The results suggest that the *dbu* operon provides an advantage for *P. putida* in environmental niches where both D-tyrosine and D-BCAAs are present.

**TABLE 2:** *P. putida* can utilize D-tyrosine when D-BCAAs are provided^*a*^

Nitrogen source(s)	Final OD_500_
Ammonium chloride (10 mM)	1.5 ± 0.05
D-tyrosine (5 mM)	0.26 ± 0.05
D-tyrosine (5 mM), D-leucine (125 µM)	1.1 ± 0.22
D-tyrosine (5 mM), D-isoleucine (125 µM)	1.7 ± 0.14
D-tyrosine (5 mM), D-valine (125 µM)	1.59 ± 0.15
D-leucine (125 µM)	0.19 ± 0.01
D-isoleucine (125 µM)	0.18 ± 0.01
D-valine (125 µM)	0.18 ± 0.01

^*a*^ Growth yield was OD_500_ after 72 h incubation in M8 salts with glucose (11 mM) as the carbon source and the indicated nitrogen source(s). Ammonium chloride (10 mM), D-tyrosine (5 mM), and/or D-BCAAs (125 μM) were in the growth medium. Results are the mean and standard deviation between three independent biological replicates. Statistical significance was determined by Sidak’s multiple comparisons test. Addition of D-BCAAs to the medium with D-tyrosine significantly (*P* < 0.05) increased final yield compared to both the medium with only D-tyrosine or only another BCAA.

### Conclusions

Results herein support the conclusions that (i) DbuR is a transcriptional repressor of the *dbu* operon, (ii) at least three D-BCAAs serve as a signal to allow transcription of the *dbu* genes, seemingly in a DbuR-independent manner, and (iii) increased expression of the *dbu* genes allows *P. putida* to utilize D-tyrosine as a nitrogen source.

DbuR binds a sequence upstream of the *dbuA* coding sequence, which includes a putative 20 bp palindrome and the −10 element of the *dbu* promoter (Fig. 3). DbuR binding would therefore be expected to prevent the binding of RNA polymerase, consistent with low transcription of the operon when DbuR is present. Efforts in this study did not identify the effector(s) of DbuR that would release repression of the *dbu* operon. Because *dbu* genes were not functional in the expression studies herein, it is possible that the effector(s) are a metabolic intermediate in the catabolism of one or more D-BCAAs. This caveat eliminated our ability to identify a metabolite of this pathway as a potential effector, and additional studies are needed to define the presumed effector of DbuR.

Beyond the clear role of DbuR in controlling transcription of the *dbu* operon, at least three D-BCAAs induced its expression, seemingly in a DbuR-independent manner. These data suggested a model in which an activator binds in the presence of each of at least D-isoleucine, D-leucine, or D-valine. In this scenario, when bound to an effector, the activator would outcompete or otherwise prevent the binding of DbuR and activate transcription to a level dependent on the specific amino acid. Initial mutant screens failed to identify a plausible activator. Additional studies will be required to define the mechanism used by the D-BCAAs to induce *dbu* transcription.

**Fig 6:**
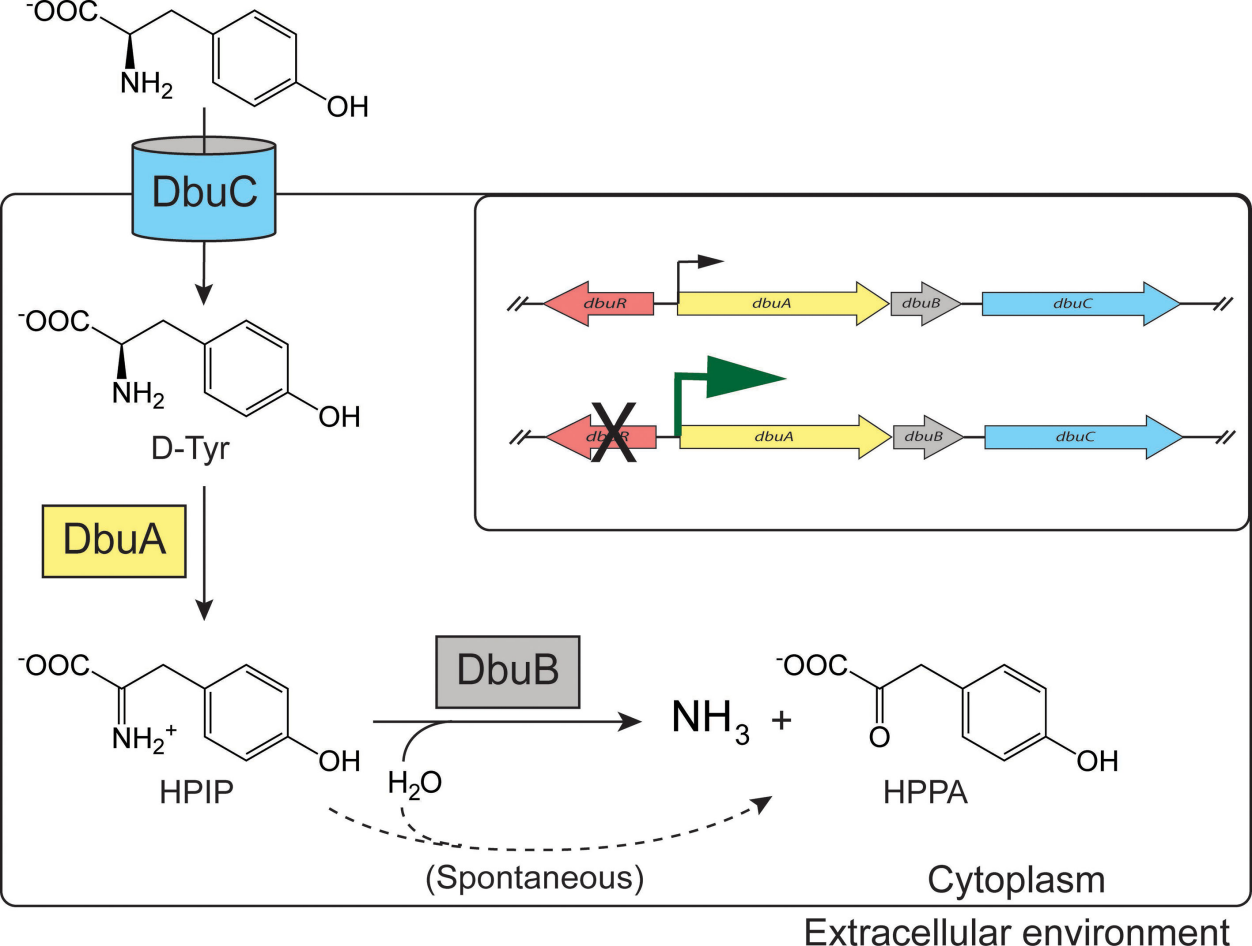
Role of Dbu proteins in D-tyrosine catabolism in *P. putida*. The gene products of the *dbu* operon contribute to D-tyrosine catabolism in the following ways: DbuC imports D-tyrosine, which is oxidized by DbuA to generate the imine, hydroxyphenyliminopropionate (HPIP). HPIP is then deaminated either spontaneously by water or, more rapidly, by the Rid2 protein DbuB, which generates ammonia and HPPA. The inset shows the *dbu* locus, emphasizing that in the presence of *dbuR,* expression of the *dbu* operon is too low to allow catabolism of D-tyrosine. When *dbuR* is inactivated, high expression of *dbuABC* results in catabolism of D-tyrosine sufficient to provide nitrogen for growth.

D-tyrosine did not induce *dbu* transcription like other D-BCAAs. However, the *dbu* gene products can allow for the utilization of D-tyrosine as a sole nitrogen source in the absence of DbuR, or when the *dbu* genes are overexpressed by another mechanism, such as the presence of D-BCAAs. These results may indicate a lesser role for the *dbu* genes in D-tyrosine utilization compared to D-BCAAs, either due to metabolic potential or environmental availability. The metabolic fate of D-tyrosine is schematically shown in Fig. 6. The end products of this pathway are predicted to be ammonia and 4-hydroxyphenylpyruvic acid (HPPA). HPPA is an intermediate in tyrosine biosynthesis (17) and may contribute to the biosynthesis of L-tyrosine, analogous to what was shown for D-leucine, D-valine, and D-isoleucine catabolized by the *dbu* pathway (8). This outcome could be useful for *P. putida*, as there is no annotated racemase that would convert D-tyrosine to the L-tyrosine needed for biomass. Additionally, HPPA could enter the homogentisate pathway, where it would be converted to fumarate and acetoacetate to be funneled to central metabolism (18). The catabolism of D-tyrosine might provide a fitness advantage in environments where it is present, such as when bacteria are living in association with plant or animal hosts, and may provide a benefit when D- but not L-tyrosine is available (19–21). Further analysis is required to better identify the physiologically relevant role of D-tyrosine catabolism by the *dbu* gene products, and how/if the operon is regulated in response to this role. A regulatory strategy might exist that allows *P. putida* to utilize an alternative nitrogen source, while catabolizing D-tyrosine as a side process (22–24).

D-tyrosine catabolism by *P. putida* could have industrial applications, given the utility of this organism as a chassis for diverse bioengineering and bioremediation efforts. For example, tyrosine and tyrosine-derived compounds can be used as a partial starting material for the production of biopolymers and sustainable bioplastics (25, 26). Additionally, D-tyrosine and other D-amino acids, such as D-BCAAs, can be present in wastewater and other protein-rich waste as a byproduct of microbial activity breaking down larger peptides and/or aromatic compounds present (27–29). Therefore, the ability of *P. putida* to utilize D-tyrosine, especially in the presence of D-BCAAs, could be leveraged to engineer this organism to better detoxify these wastes with the potential to generate sustainable biopolymers from the aromatic compounds present therein. In addition to the bioremediation and the production of biopolymers, D-tyrosine utilization by *P. putida* could have applications in the production of other tyrosine-derived industrial compounds, such as food additives, pharmaceuticals, and other economically relevant chemicals (30). In total, the data herein expand our understanding of the metabolic capabilities of *P. putida* from a basic science perspective and set a foundation for future bioengineering efforts using this robust and industrially relevant bacterium.

In total, the work herein defined a regulatory framework for the *dbu* operon and characterized a new metabolic capability of *P. putida*. Furthermore, the ability of *P. putida* to use D-tyrosine as a nitrogen source only in the presence of D-BCAAs emphasizes that metabolic capacities available in complex nutrient environments could be overlooked in standard laboratory conditions. This study lays the foundation for additional and expanded physiological and mechanistic characterization of a generalized catabolic pathway for D-BCAAs in *P. putida.*

## MATERIALS AND METHODS

### Cultivation, media, and chemicals

Lysogeny broth (LB; 10 g/L tryptone, 5 g/L yeast extract, 5 g/L NaCl) was used as a rich medium. Unless otherwise indicated, all strains were cultured in LB, with *P. putida* strains incubated at 30°C and *Escherichia coli* strains at 37°C. Medium for the growth of *E. coli* RHO5 included 2,6-diaminopimelic acid (1 mM). M8 minimal salts (12 mM Na_2_HPO_4_, 22 mM KH_2_PO_4_, 1.0 mM NaCl, 0.1 mM MgSO_4_) were used as minimal medium and included trace minerals (31) in addition to respective carbon and nitrogen sources. Glucose (11 mM), ammonium chloride (10 mM) were present, and D-amino acids were added as a carbon source (20 mM) or as a nitrogen source (5 mM), or as indicated. Terrific broth (TB; 12 g/L tryptone, 24 g/L yeast extract, 4 mL/L glycerol, 17 mM KH_2_PO_4_, 72 mM K_2_HPO_4_) was used as growth medium for protein overexpression. Unless otherwise stated, gentamycin (Gm) was at 20 µg/mL (*E. coli*) or 50 µg/mL (*P. putida*), kanamycin (Km) was at 50 µg/mL, and ampicillin (Ap) was at 150 µg/mL. All chemicals were purchased from Millipore Sigma (St. Louis, MO).

### Molecular techniques

Q5 DNA polymerase, purchased from New England Biosciences (NEB, Ipswich, MA), was used to amplify DNA by polymerase chain reaction (PCR) for cloning. PCR products and restriction endonuclease-digested plasmids used for cloning were purified by gel extraction using the QIAquick gel extraction kit (Qiagen; Germantown, MD). Standard cloning procedures were used to construct plasmids with appropriate inserts. Gibson assemblies were performed using NEBuilder HiFi DNA Assembly Master Mix (NEB) to generate constructs with ≥2 inserts (32). Restriction endonucleases and appropriate buffers were purchased from NEB. Plasmids were purified using the GeneJet Plasmid Miniprep kit from ThermoFisher Scientific (Waltham, MA). Quick-load Taq 2X Master Mix (NEB) was used for colony PCR. Oligonucleotides were purchased from Eton Bioscience Inc. (San Diego, CA) except for 6-FAM and HEX-labeled oligonucleotides, which were purchased from Eurofins Genomics (Louisville, KY). Sanger sequencing was performed by Eurofins Genomics. Oligonucleotides used are listed in Table S1.

Plasmids for the chromosomal deletion or replacement of genes in *P. putida* were generated by assembling 800 bp flanking the region of interest, along with the insert intended for chromosomal replacement, into the EcoRI and SalI sites of pK18msB. Gene deletions and replacements were confirmed by colony PCR and Sanger sequencing. For pJM220-derived plasmids, inserts were cloned into the PstI and HindIII sites of pJM220. pTEV19-DbuR was generated by cloning DbuR into the BspQI sites of pTEV19 (33).

### Strain construction

All strains and plasmids used in this study are listed in Table 3. Deletion mutants of *P. putida* were generated by allelic exchange via selection/counterselection as described previously (34). In short, 800 bp regions upstream and downstream of the gene of interest were cloned into EcoRI and SacI sites of the pK18msB suicide plasmid. The resulting plasmids were electroporated into the *P. putida* parent strain, and transformants were plated onto LB with 50 µg/mL of kanamycin (LB Km50) to select for merodiploids that had the plasmid integrated into the chromosome. Merodiploids were streaked for isolation on LB Km50, then onto TYS25 medium to select for a second recombination event that excises the plasmid backbone from the chromosome. Sucrose-resistant colonies were screened by colony PCR to confirm that the gene of interest was deleted. Chromosomal replacement of *dbuA* with *lacZ* was performed in a similar fashion, with *lacZ* flanked by 800 bp of homology upstream and downstream of *dbuA* cloned into pK18msB. The resulting plasmid was electroporated into *P. putida* and strains screened as described.

**TABLE 3:** Strains and plasmids used in this study

Plasmid	Description	Source
pTNS3	Tn7 helper plasmid	(35)
pK18msB-*dbuA*::*lacZ*	pK18msB with *lacZ* flanked by 800 bp of homology flanking *dbuA* cloned into EcoRI and SalI sites	This study
pTEV19-*dbuR*	pTEV19 with *dbuR* cloned into BspQI sites	This study
pJM220	pUC18T-miniTn7T-Gm-*rhaSR-PrhaBAD*	(36)
pTn7-Gm	pUC18T-mini-Tn7T-Gm	(36)
pTn7R6K-Km	pUC18R6KT-mini-Tn7T-Gm	(36)

Strain	Genotype	Source
*P. putida*	Derivatives of strain KT2440	
DMPA 26	Δ*dbuB* (*PP_2247*)	(8)
DMPA 28	Wild type	Downs Laboratory
DMPA 73	Δ*dbuA* (*PP_2246*)	(8)
DMPA 87	*ΔdbuR* (*PP_2245*)	(8)
DMPA 88	Δ*dbuC* (*PP_2248*)	(8)
DMPA 130	Wild-type *att*Tn7::*rhaSR-PrhaBAD*-empty	This study
DMPA 164	*ΔdbuR ΔdbuA*	This study
DMPA 165	*ΔdbuR ΔdbuB*	This study
DMPA 166	*ΔdbuR ΔdbuC*	This study
DMPA 167	*att*Tn7::*rhaSR-PrhaBAD-dbuA*	This study
DMPA 168	*att*Tn7::*rhaSR-PrhaBAD-dbuC*	This study
DMPA 194	*ΔdbuR att*Tn7::*rhaSR-PrhaBAD*-empty	This study
DMPA 195	*ΔdbuR att*Tn7::*rhaSR-PrhaBAD-dbuR*	This study
DMPA 199	*ΔdbuR att*Tn7::Gm^R^	This study
DMPA 219	*att*Tn7::*rhaSR-PrhaBAD-dbuAB*	This study
DMPA 220	*att*Tn7::*rhaSR-PrhaBAD-dbuBC*	This study
DMPA 221	*att*Tn7::*rhaSR-PrhaBAD-dbuAC*	This study
DMPA 222	*att*Tn7::*rhaSR-PrhaBAD-dbuABC*	This study
DMPA 223	*dbuA*::*lacZ*	This study
DMPA 224	*ΔdbuR dbuA*::*lacZ*	This study
DMPA 231	*ΔdbuR ΔdbuB att*Tn7::Gm^R^	This study
DMPA 232	*ΔdbuR ΔdbuB att*Tn7::Km^R^	This study
*E. coli*		
NEB5a	*fhuA2::IS2* Δ(*mmuPmhpD*)*169 rfbD1 luxS11rph*^WT^	Downs Laboratory
BL21-AI	*ompT gal dcm lon hsdS_B_*(*r_B_m_B_*)[*malB*^+^]_K12_(λ^S^)*araB*::*T7RNAP-tetA*	Downs Laboratory
RHO5	Δ*asd*::*FRT* Δ*aphA*::*FRT attTn7*::*pir-116-FRT*	(37)

Insertion of mini-Tn7 cassettes into the *att*Tn7 site of *P. putida* was performed as described previously (35, 38). Plasmids were introduced into recipient strains of *P. putida* via puddle mating with *E. coli* RHO5 carrying the mini-Tn7 delivery plasmid and *E. coli* RHO5 carrying pTNS3. Tn7 delivery plasmids were either pTn7R6K-Km, pTn7-Gm, or derivatives of pJM220 with the relevant gene cloned into PtsI and HindIII sites. Transconjugants were selected for on either LB Km50 (for pTn7R6K-Km) or LB with 50 µg/mL of gentamicin (LB Gm50; for pTn7-Gm and pJM220 derivatives). Insertion into the *att*Tn7 site was confirmed by colony PCR.

### Growth analyses

Growth of *P. putida* strains was measured with optical density readings at 500 nm (OD_500_) in 96-well plates (Falcon) using a FluorSTAR Omega plate reader (BMG Biotech). Cultures were grown at 30°C with orbital shaking at 200 rpm unless otherwise stated. Overnight cultures of *P. putida* were grown in LB (1 mL), pelleted, and resuspended in 2 mL of sterile saline. A 5 µL of cell suspension was used to inoculate 195 µL of each indicated medium. GraphPad Prism version 10.2.1 was used for data plotting and statistical analyses.

### Purification of DbuR

Thirty milliliters of *E. coli* BL21-AI harboring pTEV19-DbuR was grown overnight at 37°C in TB supplemented with 150 µg/mL ampicillin (TB Ap150). Fifteen milliliters of the overnight culture was used to inoculate each of two baffle-bottom Fernbach flasks containing 1.5 L of TB Ap150. The resulting cultures were grown at 37°C with shaking at 135 rpm to mid-exponential phase (OD_650_ = 0.5), at which time expression of *dbuR* was induced with the addition of 0.2% (wt/vol) arabinose and 0.2 mM IPTG. After induction, cultures were grown for 18 h at 23°C before being harvested by centrifugation at 6,000 × *g* for 20 min. Cell pellets were resuspended in 3 mL/g cells (wet weight) of binding buffer (50 mM HEPES, 150 mM NaCl, 5 mM imidazole, 10% glycerol, pH 7.5) supplemented with 2 mg/mL lysozyme, 0.2 mg/mL DNase, and 1 mM permethylsulfonyl fluoride. Following a 45 min incubation on ice, cells were mechanically lysed using a Constant Systems One Shot (United Kingdom) at 20 kpsi. Lysates were centrifuged at 45,000 × *g* for 20 min and filtered using a 0.45 µm syringe-driven filter before being loaded onto a gravity column (2.5 × 10 cm) containing 4 mL HisPur Ni-NTA Superflow Agarose (ThermoFisher Scientific). Five column volumes (CV) of wash buffer (50 mM HEPES, 150 mM NaCl, 20 mM imidazole, 10% glycerol, pH 7.5) were passed over the column. Bound protein was eluted with 5 CV of elution buffer (50 mM HEPES, 150 mM NaCl, 250 mM imidazole, 10% glycerol, pH 7.5). Cleavage of the N-terminal MBP-His6 tag (encoded by pTEV19) was performed by adding recombinant TEV protease (rTEV) to the eluted protein at a 1:50 ratio (rTEV:protein), and the sample was dialyzed against 3 L of storage buffer (50 mM HEPES, 150 mM NaCl, 10% glycerol, pH 7.5) overnight at 4°C. TEV cleavage reactions were passed over a gravity column containing Ni-NTA resin (ThermoFisher Scientific; 4 mL CV) and a gravity column containing Amylose Resin (NEB; 5 mL CV) to separate DbuR from un-cleaved and non-specifically bound proteins remaining. Eluate from this column was dialyzed against 1 L of storage buffer for 30 min at room temperature before being moved to a fresh 1 L of storage buffer and dialyzed for another 30 min at room temperature. Protein concentration was determined spectrophotometrically using a NanoDrop 2000 (ThermoFisher Scientific) along with the molecular weight (21.6 kDa) and molar extinction coefficient (11,920 M^−1^cm^−1^) of DbuR. The molar extinction coefficient was based on the sequence of DbuR purified from pTEV19, which leaves a Gly-Phe linker at the N-terminus, following cleavage by TEV protease. Proteins were separated by electrophoresis (SDS-PAGE) before staining the gel with Coomassie Blue and imaged using an Axygen GD1000 Gel Documentation System (Axygen; Union City, CA). Purity of DbuR was determined to be >95% by densitometry (Fig. S2) using VisionWorks software version 8.22.18309.10577. Purified protein was concentrated using an Amicon-Ultra-15 centrifugal filter (Millipore) with a molecular weight cutoff of 10 kDa. Purified DbuR was flash-frozen using liquid nitrogen and stored at −80°C until use.

### β-galactosidase assays

β-galactosidase activity was assayed using a FluoSTAR Omega plate reader using a one-step method described previously (39). Briefly, strains were grown overnight in 2 mL of LB at 30°C with shaking, pelleted, and resuspended in 2 mL sterile saline. Cell suspensions (75 µL) were inoculated with 3 mL of M9 minimal medium (11 mM glucose, 10 mM ammonium chloride) with the indicated supplements. Cultures were grown at 30°C with shaking to the exponential phase (OD_600_ = 0.3–0.5) before assaying β-galactosidase activity. Reactions (200 µL) were initiated by adding 80 µL cells to 120 µL of β-gal Mix (Na_2_HPO_4_ [60 mM], NaH_2_PO_4_ [40 mM], KCl [10 mM], MgSO_4_ [1 mM], 2-mercaptoethanol [40 mM], ortho-nitrophenyl-β-galactoside [3 mM], lysozyme [0.17 mg/mL], PopCulture Reagent [6.7% vol/vol], pH 7.0). The absorbance at 420 nm was measured every 60 s for 30 min at 30°C with double orbital shaking at 250 rpm between measurements. Rates (ΔA_420_/min) were taken in the linear range for each sample and corrected by subtracting rates measured in reactions containing sterile growth medium instead of cells for each condition. Miller Units were calculated using the following formula:

$Miller\;Units=\frac{1,000\;\times \;\left({OD}_{420}/min\right)}{initial\;culture\;{OD}_{600}\;\times \;vol.\;cells\;added\;\left(mL\right)}$ (28, 29, 40).

### Electrophoretic mobility shift assays

EMSA reactions (15 µL) contained HEPES (50 mM), NaCl (150 mM), glycerol (10%, vol/vol), and indicated concentrations of probe and DbuR protein. A 0.5× Tris-boric acid-EDTA (TBE) polyacrylamide (7.5%) gel was pre-developed at 75 V for 45 min at 4°C in 0.5× TBE prior to setting up each reaction. To quantify DbuR binding to the promoter region of the *dbu* locus, purified DbuR (0, 20, 40, 100, 200, or 400 nM) was incubated with 20 nM of probe, which was *Pdbu* (133 bp) with a 5′-6-carboxyfluorescein (6-FAM) label. Specificity of DbuR binding was determined by incubating 200 nM DbuR with 20 nM of 6-FAM-labeled *Pdbu*, along with a gradient of concentrations of unlabeled (cold) *Pdbu*. All reactions were carried out at room temperature for 15 min. Three microliters of 50% glycerol (vol/vol) was added to reactions before 7.5 µL of each reaction was resolved on a polyacrylamide gel at 75 V in 0.5× TBE at 4°C for 2.5 h. Dye containing xylene cyanol and bromophenol blue was loaded into a control well as an indicator of sample migration. Gels were imaged using a Typhoon Trio Imager (GE Healthcare) with excitation at 525 nm and the 488 (Blue) filter.

### DNase I footprinting

DNase I footprinting was performed as described previously (41, 42). Briefly, a probe was generated by amplifying the promoter region upstream of the *dbu* locus (133 bp between *dbuR* and *dbuA*) by PCR using oligonucleotides with 5′-6FAM (forward) and 5′-hexachlorofluorescein (HEX, reverse) labels. The PCR product was purified by gel extraction. A 150 nM probe was incubated in the presence or absence of 1.5 µM DbuR in 250 µL of EMSA buffer (50 mM HEPES, 150 mM NaCl, 10% glycerol), at room temperature for 40 min, followed by the addition of 25 ng of DNase I. Reactions were incubated at room temperature for 5 min prior to heat-inactivation at 80°C for 20 min. Reactions were purified using the QAIquick PCR purification kit (Qiagen) and eluted with 30 µL of nuclease-free water. Fragment analysis was performed by Eton Biosciences using the Applied Biosystems 3730xl DNA Analyzer. Results from fragment analysis were analyzed using GeneMapper 6 software and compared to results from Sanger sequencing.

### Sanger sequencing

The 133 bp probe, containing the promoter region of the *dbu* locus, was used as the template for Sanger sequencing. The template was sequenced using the USB ThermoSequenase Cycle Sequencing Kit (Affymetrix) using HEX and 6FAM labeled oligonucleotides to generate the digested fragment. Fragment analysis was performed by Eton Biosciences using the Applied Biosystems 3730xl DNA Analyzer, and data were analyzed using GeneMapper 6 software.

## Data Availability

All relevant data are included in the content of this paper.

## SUPPLEMENTAL MATERIAL

Supplemental material aem.01715-25-s0001.pdf Table S1; Fig. S1 to S4.

ASM does not own the copyrights to Supplemental Material that may be linked to, or accessed through, an article. The authors have granted ASM a non-exclusive, world-wide license to publish the Supplemental Material files. Please contact the corresponding author directly for reuse.
