# Supplementary material for: The XRE family protein DbuR is a transcriptional repressor of the dbu operon in Pseudomonas putida
Source: Appl Environ Microbiol. 2025 Nov 25;91(12):e01715-25. doi: 10.1128/aem.01715-25 (PMC12724383; doi:10.1128/aem.01715-25)
Supplement: Supplemental material — Table S1; Fig. S1 to S4. [file aem.01715-25-s0001.pdf]

SUPPLEMENTAL MATERIAL

The XRE family protein DbuR is a transcriptional repressor of the *dbu* operon in *Pseudomonas putida*

Ronnie L. Fulton[1], Diana M. Downs[*]
Department of Microbiology
University of Georgia, Athens, GA, USA.

Table S1: Oligonucleotides used in this study.

Oligonucleotide	Sequence
T7-F	TAATACGACTCACTATAGGG
T7-R	GCTAGTTATTGCTCAGCGG
M13 F	GTAAAACGACGGCCAGT
M13-R	CAGGAAACAGCTATGAC
ppu-glmS-up	CACCATTCCGCTGCAGTTG
ppu-glmS-down	AGTCAGAGTTACGGAATTGTAGG
pTn7-R	CACAGCATAACTGGACTGATTTC
pTn7-L	ATTAGCTTACGACGCTACACCC
Tn7-end	GGGGTGGAAATGGAGTTTTT
oJM730	GATACAGCGTGAATTTTCAGG
ppu-K18-2245-USF	acagctatgacatgattacgCCGCTACCAAGGTCAGTG
ppu-K18-2245-USR	ggtgcgcaagATCTACCTGTCGGAAAGC
ppu-K18-2245-DSF	acaggtagatCTTGCGCACCTGGCGCAG
ppu-K18-2245-DSR	caagcttgcatgcctgcaggCGGTCCGCCCAGGCACCT
ppu-2245-seq-F	TCAACGATCGGTCATCACTACCATG
ppu-2245-seq-R	ATGCCCGCAGCTCTTCCC
ppu-K18-2246-USF	ggatccccgggtaccgagctAGGCGCAGCGGATCAACG
ppu-K18-2246-USR	ctcctcgggcGCGCGAAAGTTTGCTCAAGC
ppu-K18-2246-DSF	actttcgcgcGCCCGAGGAGTTCGACGT
ppu-K18-2246-DSR	acagctatgacatgattacgGGCCGCAGATGGCCAAAATC
ppu-2246-seq-F	GCGGGCATAGGCCGGTGTCA
ppu-2246-seq-R	GCGGCCTTGCAGCCCATCGC
ppu-K18-2247-USF	ggatccccgggtaccgagctACGAAGCGCCACCGCAGG
ppu-K18-2247-USR	gtacacctcgGCCTTTACCGCGCGGAAAAC
ppu-K18-2247-DSF	cggtaaaggcCGAGGTGTACAAGGCGTTC
ppu-K18-2247-DSR	acagctatgacatgattacgGTGGTGACACTTCGTTGG
ppu-2247-seq-F	GCCTTCACCTTCCCTGGTCC
ppu-2247-seq-R	GCACACGATACCGACCACC
ppu-K18-2248-USF	acagctatgacatgattacgGTTTTTCTGGCTGGCGGCCCAGG
ppu-K18-2248-USR	ccaggctacaGGCGCTGACCAGCGCGCC
ppu-K18-2248-DSF	ggtcagcgccTGTAGCCTGGTGGGGTTC
ppu-K18-2248-DSR	caagcttgcatgcctgcaggGTGGTTGAAGAAGCCATCAG
ppu-2248-seq-F	ATGCAAGAGCAGCTGAAGATCG
ppu-2248-seq-R	TTATGCCGCCACCGCCTTC
ppu-JM220-dbuR-F	aatgaaattcaactagtgctctgcaATGCCCGCAGCTCTTCCC
ppu-JM220-dbuR-R	aggccttcgcgaggtaccgggcccaTCAACGATCGGTCATCACTACCATG
ppu-JM220-dbuB-F	aatgaaattcaactagtgctctgcaATGAGCAATGACATTCAGCGTTTCC
ppu-JM220-dbuB-R	aggccttcgcgaggtaccgggcccaTCAGGCGTCGCCGACGAA
ppu-JM220-dbuAB-F	aatgaaattcaactagtgctctgcaATGACCCCGACCTACGACAC
ppu-JM220-dbuAB-R	aggccttcgcgaggtaccgggcccaTCAGGCGTCGCCGACGAA
ppu-JM220-dbuBC-F	aatgaaattcaactagtgctctgcaATGAGCAATGACATTCAGCGTTTCC

ppu-JM220-dbuBC-R	aggccttcgcgaggtaccgggcccaTTATGCCGCCACCGCCTT
ppu-JM220-dbuAC-A-F	aatgaaattcaactagtgctctgcaATGACCCCGACCTACGAC
ppu-JM220-dbuAC-A-R	gctctttgcccggTCAGGGTTGCTGGAAACG
ppu-JM220-dbuAC-C-F	cagcaaccctgaCCGGGCAAAGAGCCCGTG
ppu-JM220-dbuAC-C-R	aggccttcgcgaggtaccgggcccaTTATGCCGCCACCGCCTTC
ppu-JM220-dbuABC-F	aatgaaattcaactagtgctctgcaATGACCCCGACCTACGAC
ppu-JM220-dbuABC-R	aggccttcgcgaggtaccgggcccaTTATGCCGCCACCGCCTT
Pdbu-EMSA-F (5’ 6 FAM)	TCAGGGATCTCGGCATGC
Pdbu-EMSA-R	TGTCGTAGGTCGGGGTCATG
dbuC-EMSA-F (5’ 6-FAM)	TAGCCTGGTGGGGTTCATC
dbuC-EMSA-R	TTATGCCGCCACCGCCTT
Pdbu-EMSA-HEX-R (5’HEX)	TGTCGTAGGTCGGGGTCATG
ppu-K18-dbuA::lacZ-USF	aggaaacagctatgacatgattacgCGGATGCCAGTCAGCATATAGATTTTTTTATAGCCAAATAG
ppu-K18-dbuA::lacZ-USR	taatcatggtcatGCACGCTCCTGCCAGGCA
ppu-K18-dbuA::lacZ-lacZ-F	gcaggagcgtgcATGACCATGATTACGGATTC
ppu-K18-dbuA::lacZ-lacZ-R	gggatgctccttTTATTTTTGACACCAGACC
ppu-K18-dbuA::lacZ-DSF	gtgtcaaaaataaAAGGAGCATCCCATGAGC
ppu-K18-dbuA::lacZ-DSR	agtgccaagcttgcatgcctgcaggCCAGCATGACCACGGTGAC
ppu-TEV19-dbuR-F	nngctcttcnttcATGCCCGCAGCTCTTCCC
ppu-TEV19-dbuR-R	nngctcttcnttaTCAACGATCGGTCATCACTACCATG
ppu-TEV19-dbuR-L28A-F	TCGGACAATTGCTTGGCGGTCATCTGGCGCGC
ppu-TEV19-dbuR-L28A-R	GCGCGCCAGATGACCGCCAAGCAATTGTCCGA
ppu-TEV19-dbuR-S40A-S43A-F	AGTTCCATCTTCGCCAACGTGGCCAGCGGCACGCC
ppu-TEV19-dbuR-S40A-S43A-R	GGCGTGCCGCTGGCCACGTTGGCGAAGATGGAACT
ppu-TEV19-dbuR-ELA46AAT-F	TTTCATAGCTGACCGACACCTGCGTCGCTGCCATCTTCGACAACGTGGACAG
ppu-TEV19-dbuR-ELA46AAT-R	CTGTCCACGTTGTCGAAGATGGCAGCGACGCAGGTGTCGGTCAGCTATGAAA
ppu-TEV19-dbuR-H137A-F	GAACTCTTGCCCGGGGGCGCGGATAAAATCGTCG
ppu-TEV19-dbuR-H137A-R	CGACGATTTTATCCGCGCCCCCGGGCAAGAGTTC
ppu-TEV19-dbuR-E141A-F	CCAGCGCGAACGCTTGCCCGGGG
ppu-TEV19-dbuR-E141A-R	CCCCGGGCAAGCGTTCGCGCTGG
ppu-TEV19-dbuR-H176A-F	CCGACAGGTAGATGGCCCCCACCTGGCTGT
ppu-TEV19-dbuR-H176A-R	ACAGCCAGGTGGGGGCCATCTACCTGTCGG
ppu-TEV19-dbuR-V192A-F	ACGATCGGTCATCACTGCCATGACATGGGCATC
ppu-TEV19-dbuR-V192A-R	GATGCCCATGTCATGGCAGTGATGACCGATCGT

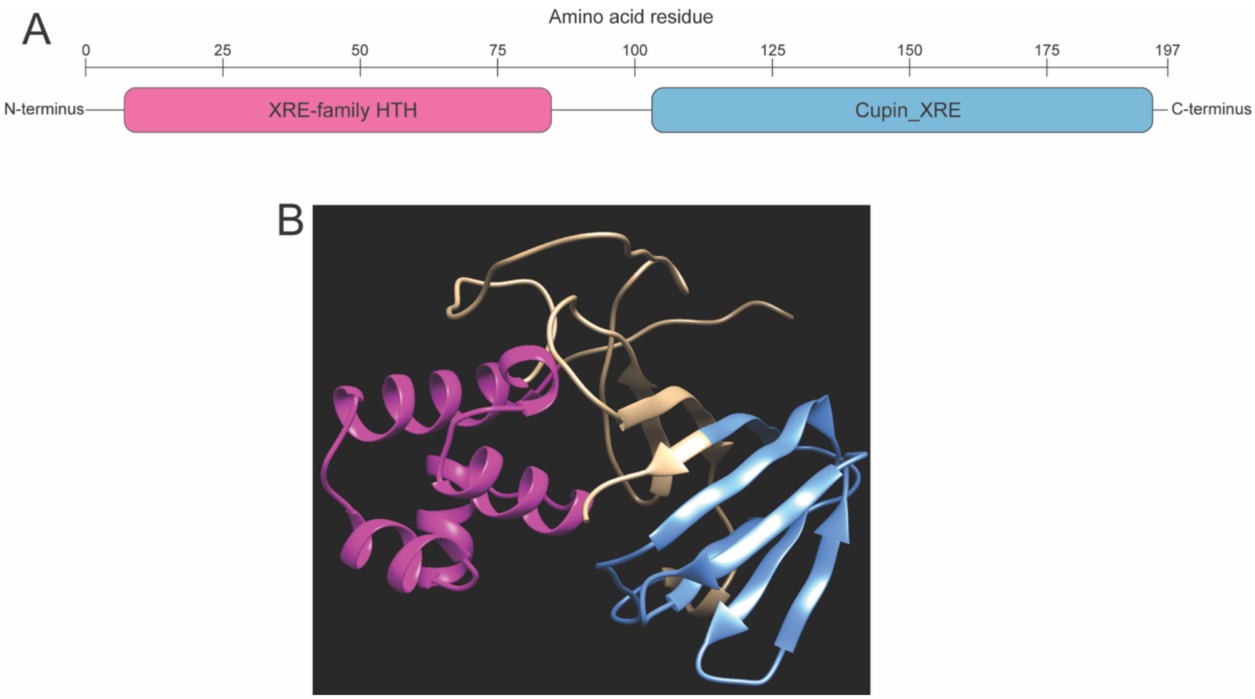

Figure S1: DbuR contains domains predicted for a XRE-family protein. Shown are the locations of the XRE-family HTH (magenta) and the Cupin_XRE (blue) domains in the primary structure of DbuR. The domains are shown in the 3-dimensional structure of DbuR with the same color scheme. Domains were predicted using NCBI Conserved Domain Database (CDD) (10, 11). The structure for DbuR was obtained from an existing AlphaFold modelled structure for the protein (AlphaFold DB Q88KP5). The image in Figure S1B was generated using Chimera version 1.11.2.

Appox. MW	22 kDa		
Purity	>95%		
Amount loaded	2 µg	4 µg	6 µg

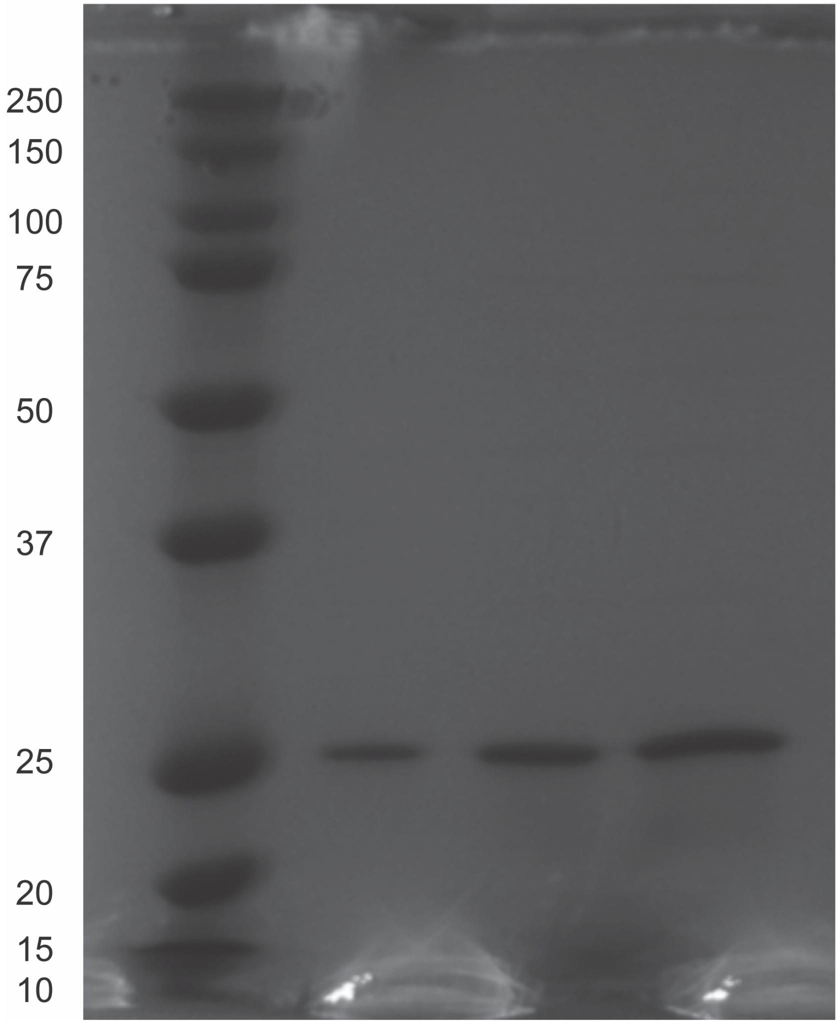

Figure S2: DbuR was purified to near homogeneity. Shown is an SDS-PAGE gel with the indicated concentrations of DbuR purified from *E.coli* and expressed from pTEV19. The gel was stained with Coomassie Blue and densitometry was performed using VisionWorks software version 8.22.18309.10577.

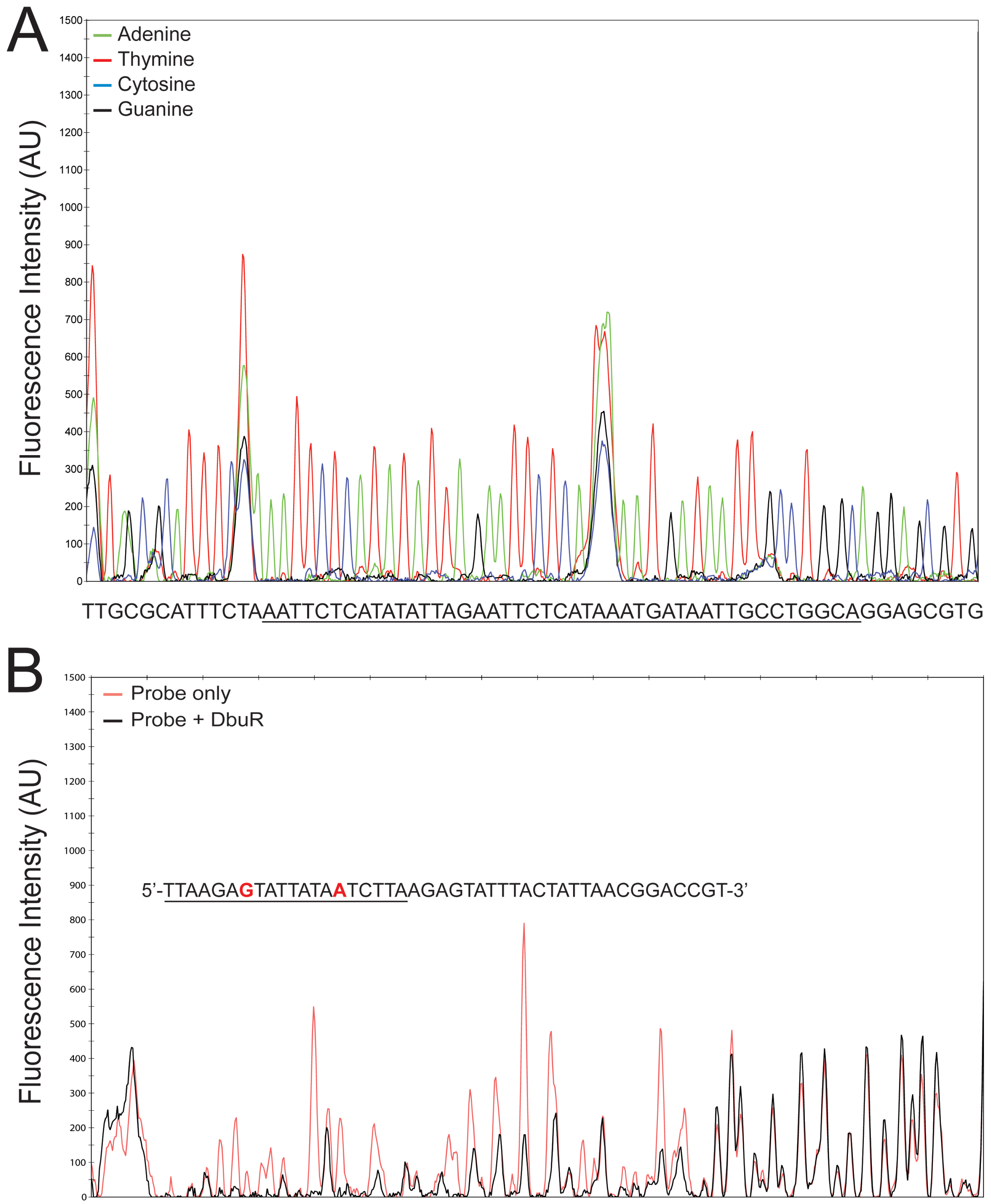

Figure S3: Alignment of peaks from (A) Sanger sequencing and (B) DNase I footprinting of the reverse (HEX-labelled) strand to be compared to that of the forward strand shown in Figure 2.

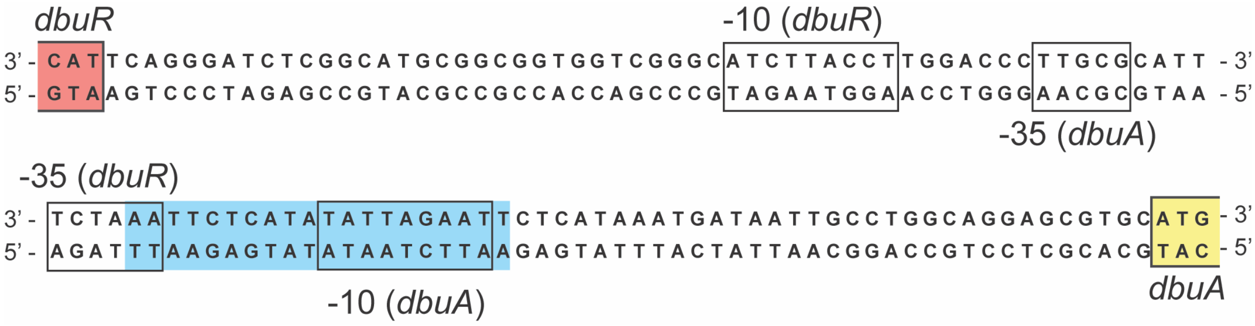

Figure S4: Expanded view of the DbuR binding site. Shown is the nucleotide sequence from the start codon of *dbuR* (red) to the start codon of *dbuA* (yellow), along with the -10 and -35 elements for each promoter and the DbuR binding site (blue). This figure is an expanded view of that shown in Figure 3.
